# Cytokine imbalance and immune network endotypes underlying heterogeneous response to omalizumab in chronic spontaneous urticaria

**DOI:** 10.3389/fimmu.2026.1783483

**Published:** 2026-04-20

**Authors:** Young-Min Ye, Hyun-Young Lee, Jae-Hyuk Jang, Da-Hye Moon, Kun-Woo Park, Semin Park

**Affiliations:** 1 Department of Allergy and Clinical Immunology, Ajou University School of Medicine, Suwon, Republic of Korea; 2 Clinical Trials Center, Ajou University Medical Center, Suwon, Republic of Korea

**Keywords:** chronic spontaneous urticaria, cytokine profiling, IL-13 (interleukin 13), omalizumab, treatment response

## Abstract

**Background:**

Chronic spontaneous urticaria (CSU) exhibits marked immunologic heterogeneity, and a substantial proportion of patients show delayed or absent responses to anti-IgE therapy. While cytokines are implicated in CSU pathophysiology, their integrated network architecture in relation to treatment response remains poorly understood.

**Methods:**

We analyzed serum cytokine profiles in 65 antihistamine-refractory CSU patients who received omalizumab for ≥ 6 months. Fifteen cytokines were quantified at baseline and month 6 using a multiplex bead-based assay. Treatment responses were categorized as early and well-controlled (EW), late or partly controlled (LP), or no-response (NR) based on UAS7 and UCT criteria. Cytokine compositional balance, network topology, multinomial regression, and structural equation modeling were applied to delineate immune endotypes associated with treatment response.

**Results:**

Of 65 patients, 33 (50.8%) were EW, 16 (24.6%) were LP, and 16 (24.6%) were NR. Among baseline cytokines, interleukin (IL)-13 was the only analyte differing significantly in absolute concentration, being higher in LP and NR than in EW patients. Compositional analyses revealed an IL-13-dominant imbalance strongly enriched in LP and NR. Cytokine-network analysis demonstrated cohesive interactions in EW, partial disruption in LP, and fragmented innate-Th17/Th1 connectivity in NR. Multinomial regression and structural path analyses identified IL-13 as a central node linking multiple inflammatory pathways associated with inadequate response. Longitudinally, meaningful cytokine modulation was observed only in EW, whereas LP and NR exhibited minimal or no changes despite treatment.

**Conclusion:**

Baseline cytokine imbalances and network architecture are closely associated with heterogeneity in omalizumab response. An IL-13–dominant, biologically rigid cytokine profile, together with persistent innate-Th17 activity in certain patients, may define CSU endotypes less amenable to anti-IgE therapy.

## 1 Introduction

Chronic spontaneous urticaria (CSU) is an inflammatory skin disease characterized by recurrent wheals, pruritus, and/or angioedema (1, 2). Achieving early and complete disease control is a key therapeutic objective in CSU (2). Persistent disease activity imposes a substantial burden, with severe CSU associated with significant impairment in quality of life, increased healthcare utilization, psychosocial distress, and prolonged disease duration (3–7).

Omalizumab, a monoclonal anti-immunoglobulin E (IgE) antibody, is an established treatment for patients with CSU who remain symptomatic despite high-dose H1-antihistamines (H1AH) (2). Clinical responses to omalizumab vary widely; fewer than one-third of patients achieve rapid and complete disease control, and a considerable proportion exhibit delayed or inadequate improvement (8, 9). This heterogeneity has prompted efforts to identify biomarkers that reliably predict treatment response.

Several clinical and serological markers—including baseline total IgE and D-dimer levels, peripheral basophil count, and indicators of autoimmune endotypes—have been evaluated; however, findings have been inconsistent (9–12). IgE autoantibodies to thyroid peroxidase (TPO) and interleukin (IL)-24, commonly detected in patients with an atopic background, have been associated with favorable responses to omalizumab; however, their assessment remains confined to research settings. Conversely, biomarkers associated with type IIb autoimmune CSU, such as immunoglobulin G (IgG) autoantibodies to TPO or thyroglobulin, autologous serum skin test (ASST) positivity, basopenia, eosinopenia, low total IgE levels, and comorbid autoimmune diseases, have demonstrated limited or variable predictive utility. Consequently, widely applicable predictors of omalizumab response remain uncertain.

Recent studies have suggested that chronic low-grade inflammation and cytokine dysregulation, particularly within T cell-mediated pathways, contribute to disease persistence and refractoriness. In previous studies, patients with H1AH-refractory CSU exhibited distinct cytokine profiles, including alterations in Th2-, Th17-, and T cell-related cytokines, compared with healthy controls or treatment-responsive patients (13–15). Considering that cytokines are central to immune signaling, local immune responses, and mast cell-driven inflammation, their integrated patterns may offer mechanistic insights and facilitate more precise treatment stratification. Nevertheless, most investigations have focused on single cytokines or absolute concentration differences, yielding inconsistent results and limited mechanistic insight. Moreover, only a small number of studies have evaluated cytokine dynamics during omalizumab treatment, often without stratification by clinical response, leaving the immunologic basis of treatment heterogeneity largely unresolved.

To address the limitations of current biomarkers, we investigated whether baseline cytokine signatures and cytokine-network characteristics are associated with early, partial, or absent responses to omalizumab in H1AH-refractory CSU. In addition, we evaluated the evolution of cytokine profiles during treatment to identify immunologic pathways that may underlie omalizumab nonresponse and inform future therapeutic strategies.

## 2 Methods

### 2.1 Study population and clinical assessments

In total, 65 patients with H1AH-refractory CSU who received omalizumab for ≥ 6 months were included. Serum samples were obtained at baseline and at 6 months, stored at ˗ 70 °C, and analyzed for cytokine levels. Disease activity and control were evaluated using the 7-day urticaria activity score (UAS7) and the urticaria control test (UCT).

Atopy was defined as ≥ 1 positive response on a skin prick test to common aeroallergens, including alder, birch, oak, grass mixture, mugwort, ragweed, cat, dog, *Dermatophagoides pteronyssinus*, *Dermatophagoides farinae*, *Aspergillus niger*, or *Alternaria* species. Total IgE was measured using ImmunoCAP (Thermo Fisher Scientific, Waltham, MA, USA). Anti-TPO and anti-thyroglobulin IgG antibodies were quantified, and the ASST was performed in accordance with the European Academy of Allergy and Clinical Immunology/Global Allergy and Asthma European Network consensus recommendations (16). Written informed consent was obtained from all the study participants.

Early response was defined as achieving a UAS7 ≤ 6 and a UCT ≥ 12 within the first month of omalizumab treatment. Well-controlled urticaria was defined by meeting the same criteria at 6 months. Patients were categorized into three groups: early and well-controlled (EW), achieving both criteria at 4 weeks and 6 months; late or partly controlled (LP), achieving both criteria at any scheduled 4-week visit after 4 weeks, or meeting only one criterion at 6 months; and nonresponders (NR), failing to achieve a UAS7 ≤ 6 or UCT ≥ 12 at any scheduled 4-week assessment during the 6-month treatment period.

### 2.2 Measurement of serum cytokines

Serum concentrations of 15 cytokines—CCL5, CCL11, interferon (IFN)-γ, IL-1β, IL-2, IL-4, IL-6, IL-8, IL-10, IL-12p70, IL-13, IL-17A, IL-33, tumor necrosis factor-α (TNF-α), and vascular endothelial growth factor—were quantified in duplicate using a Luminex-based multiplex assay (R&D Systems, Minneapolis, MN, USA). Cytokine concentrations were calculated from 5-parameter logistic standard curves ranging from 0.15 to 340,000 pg/mL. Values below the detection limit were assigned a minimal constant of 0.001 pg/mL for compositional analysis, consistent with our previous study (13).

### 2.3 Statistical analyses

Group comparisons were performed using the Mann–Whitney U test or Kruskal–Wallis test for continuous variables and Fisher’s exact test for categorical variables. Cytokine levels showed non-normal distributions and are presented as mean ± standard error. Within-subject changes in cytokine levels from baseline to month 6 were assessed using the Wilcoxon signed-rank test. To assess longitudinal changes in cytokine compositional levels according to anti-TPO IgG status, linear mixed-effects models were applied, including time, anti-TPO IgG status (with the negative group as the reference), and their interaction as fixed effects, with subject included as a random effect. Statistical analyses were performed using R (version 4.3.3; R Foundation for Statistical Computing, Vienna, Austria) and SPSS (version 29; IBM Corp. Armonk, NY, USA). Two-sided p-values < 0.05 were considered statistically significant.

#### 2.3.1 Cytokine balance analysis and heatmap visualization

To account for the compositional nature of cytokine data, baseline cytokine concentrations were transformed using the centered log-ratio (CLR) method. CLR-transformed values were expressed relative to IL-13 to characterize individual cytokine balance patterns. Heatmaps were generated using Euclidean distance and k-means clustering (k = 3) for cytokines and subjects. The distribution of EW, LP, and NR patients across clusters was compared using Fisher’s exact test.

#### 2.3.2 Exploratory graph analysis

EGA was applied to CLR-transformed cytokine data to characterize the latent cytokine network structure (17). Partial correlation networks were estimated using graphical least absolute shrinkage and selection operator with extended Bayesian information criterion model selection. Immune-cytokine communities were identified using the walktrap algorithm. In network visualizations, edge thickness represented the magnitude of correlations, and edge color indicated the direction (positive or negative) of relationships.

#### 2.3.3 Multinomial logistic regression and radar plot visualization

Multinomial logistic regression was conducted to identify baseline cytokines associated with LP or NR status compared to EW patients. Radar plots display log_10_-transformed odds ratios derived from raw baseline cytokine concentrations, using EW as the reference group. Values above or below zero indicate cytokines associated with increased or decreased odds of inadequate response, respectively.

#### 2.3.4 Structural equation modeling for pathway analysis

SEM was performed using the *lavaan* package to evaluate interrelationships among baseline cytokines and their associations with response categories. Cytokine concentrations were CLR-transformed, and total IgE was log-transformed. Model fit was assessed using the χ²/degree of freedom (χ²/df), comparative fit index (CFI), root mean square error of approximation (RMSEA), and standardized root mean square residual (SRMR). Acceptable fit thresholds were defined as χ²/df < 3, CFI ≥ 0.90, RMSEA ≤ 0.08, and SRMR ≤ 0.08. Standardized path coefficients and odds ratios were reported.

## 3 Results

### 3.1 Clinical characteristics of the study subjects

In total, 65 patients with H1AH-refractory CSU who received omalizumab for 6 months were included (**Table 1**). Of these, 33 patients (50.8%) were classified as EW, 16 (24.6%) as LP, and 16 (24.6%) as NR.

**Table 1:** Clinical characteristics of the study participants.

Variables	EW group (n = 33)	LP group (n = 16)	NR group (n = 16)	*P* value
Age (years)	42.8 ± 10.3	37.8 ± 7.9	50.1 ± 14.0	0.014
Female (%)	17 (51.5)	13 (81.3)	11 (68.8)	0.117
Urticaria duration (years)	2.1 ± 3.1	1.7 ± 2.1	2.4 ± 3.0	0.869
Atopy (%)	14/26 (53.9)	6/11 (54.6)	7/12 (58.3)	1.000
Angioedema (%)	12 (36.4)	3 (18.8)	6 (37.5)	0.448
Total IgE levels (kU/L)	166.4 ± 116.7	185.3 ± 115.7	146.4 ± 132.0	0.361
Low IgE(<40 kU/L, %)	4 (12.1)	0 (0)	3 (18.8)	0.250
Baseline UAS7	23.1 ± 8.9	28.8 ± 9.7	33.4 ± 7.2	0.002
Baseline UAS7 ≥ 28	11 (33.3)	9 (56.3)	13 (81.3)	0.006
Baseline UCT	5.1 ± 3.0	4.8 ± 3.4	3.8 ± 5.7	0.611
ASST positivity (%)	13/29 (44.8)	4/15 (26.7)	6/10 (60.0)	0.228
NECU (%)	4/18 (22.2)	2/11 (18.2)	1/11 (9.1)	0.868
IgE to HDM positivity (%)	21 (63.6)	10 (62.5)	8 (50.0)	0.715
ESR (mm/hr)	8.1 ± 4.5	9.5 ± 7.0	14.2 ± 8.5	0.062
CRP (mg/dL)	0.1 ± 0.1	0.4 ± 0.8	0.3 ± 0.5	0.175
C3 (mg/dL)	118.3 ± 17.1	122.9 ± 21.6	121.6 ± 17.6	0.820
C4 (mg/dL)	27.7 ± 8.0	28.6 ± 8.9	30.9 ± 6.8	0.427
WBC (10^3^/µL)	7.3 ± 2.2	8.6 ± 2.0	6.3 ± 1.9	0.010
Eosinophil differential (%)	1.82 ± 1.6	1.39 ± 1.5	2.01 ± 1.4	0.106
Basophil differential (%)	0.4 ± 0.2	0.4 ± 0.3	0.4 ± 0.3	0.716
Anti-TG IgG positivity (n, %)	5/23 (21.7)	6/13 (46.2)	4/13 (30.8)	0.300
Anti-TPO IgG positivity (n, %)	7/23 (30.4)	4/13 (30.8)	5/13 (38.5)	0.925

CSU, chronic spontaneous urticaria; EW, early and well-controlled; LP, late or partly controlled; NR, non-responder; UAS7, urticaria activity score over 7 days; UCT, urticaria control test; ASST, autologous serum skin test; NECU, non-steroidal anti-inflammatory drug–exacerbated chronic urticaria; HDM, house dust mite; ESR, erythrocyte sedimentation rate; CRP, C-reactive protein; WBC, white blood cell count; TG, thyroglobulin; TPO, thyroid peroxidase.

*P*-values were obtained by Fisher’s exact test or Mann-Whitney U-test.

NR patients were older than EW and LP patients (*P* = 0.014). Baseline UAS7 was highest in NR patients (33.4 ± 7.2), and the proportion of patients with severe CSU (UAS7 ≥ 28) was greater in the NR group (81.3%) compared to the EW group (33.3%, *P* = 0.006). Baseline UCT scores were similar across the three groups.

Other clinical variables—including sex, disease duration, atopy, angioedema, total IgE levels, thyroid autoantibodies, ASST positivity, NSAID-exacerbated CSU, house dust mite sensitization, erythrocyte sedimentation rate, C-reactive protein levels, complement levels, and eosinophil counts—were comparable across groups. Total white blood cell counts differed significantly (*P* = 0.010), whereas leukocyte subsets remained similar.

### 3.2 Cytokine balance relative to IL-13

At baseline, most cytokines did not differ significantly among groups in absolute concentrations, except for IL-13, which was higher in LP and NR patients than in EW patients (**Supplementary Table 1**). Because raw cytokine values offered limited discriminatory power, subsequent analyses focused on cytokine balance patterns using IL-13 as a compositional anchor.

**Figure 1:**
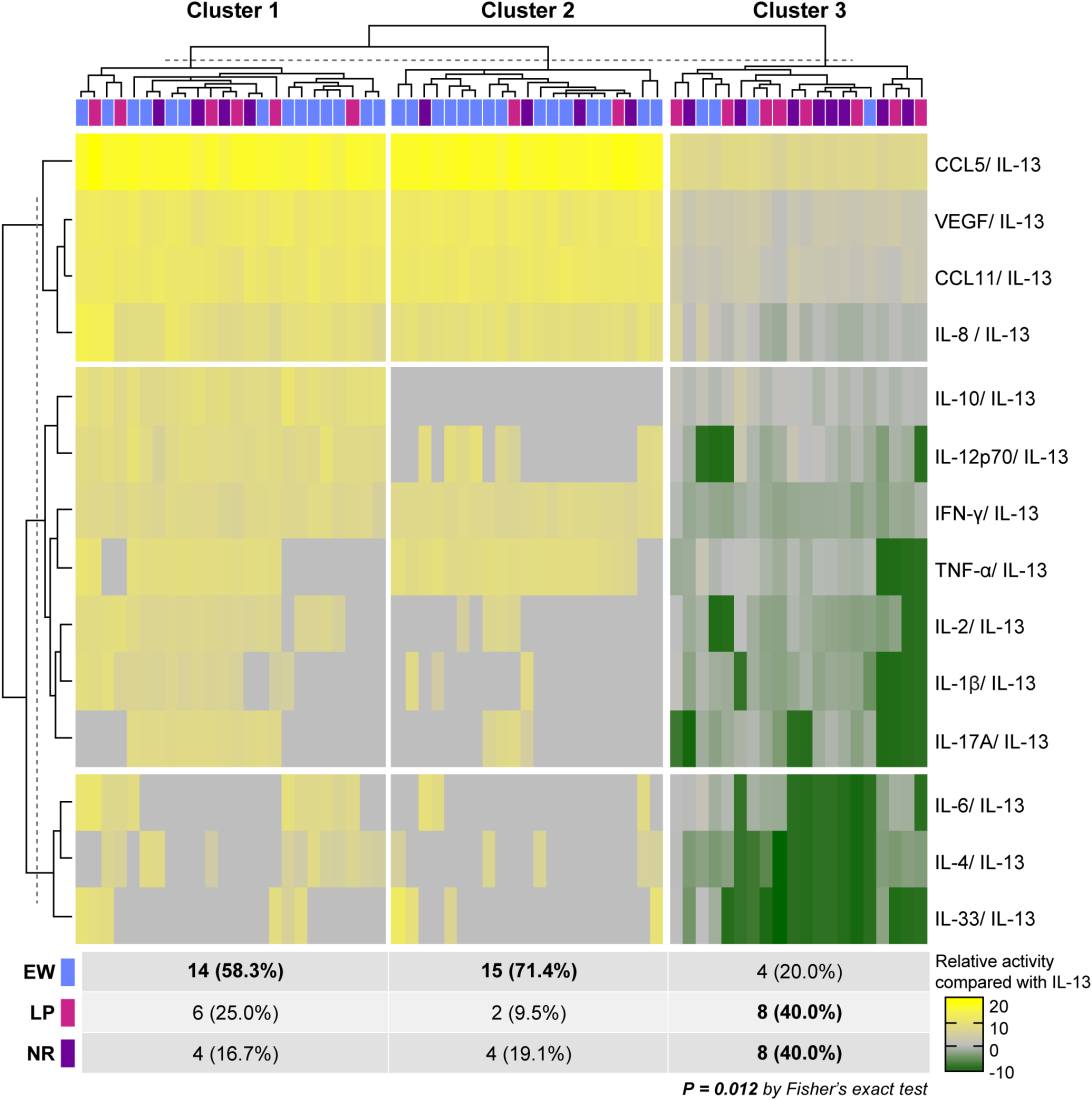
Heatmap of baseline cytokine balance relative to interleukin (IL)-13 across omalizumab response groups. Cytokine concentrations were transformed using the centered log-ratio (CLR) method and expressed as log-ratios relative to IL-13. Colors indicate lower, similar, or higher cytokine levels compared to IL-13 (green–grey–yellow). Unsupervised k-means clustering (k = 3) was applied to both cytokines (rows) and subjects (columns). The distribution of clinical response groups across clusters was assessed using Fisher’s exact test.

The cytokine-to-IL-13 heatmap revealed three distinct clusters (**Figure 1**). Cluster 1 showed relatively higher cytokine-to-IL-13 ratios and was predominantly composed of EW patients. Cluster 2 exhibited a mixed cytokine balance pattern without a clear dominance of individual cytokines relative to IL-13 but with comparatively reduced regulatory cytokine signals, including IL-10 and IL-12p70, and contained patients across all response groups. Cluster 3 was characterized by significantly lower levels of IL-4, IL-6, and IL-33 relative to IL-13, representing an IL-13–dominant pattern. This cluster was strongly enriched in poor responders: only 12.1% of EW patients were classified in Cluster 3, compared to 50% of LP and NR patients (*P* = 0.012).

### 3.3 Exploratory graph analysis of baseline cytokine networks

**Figure 2:**
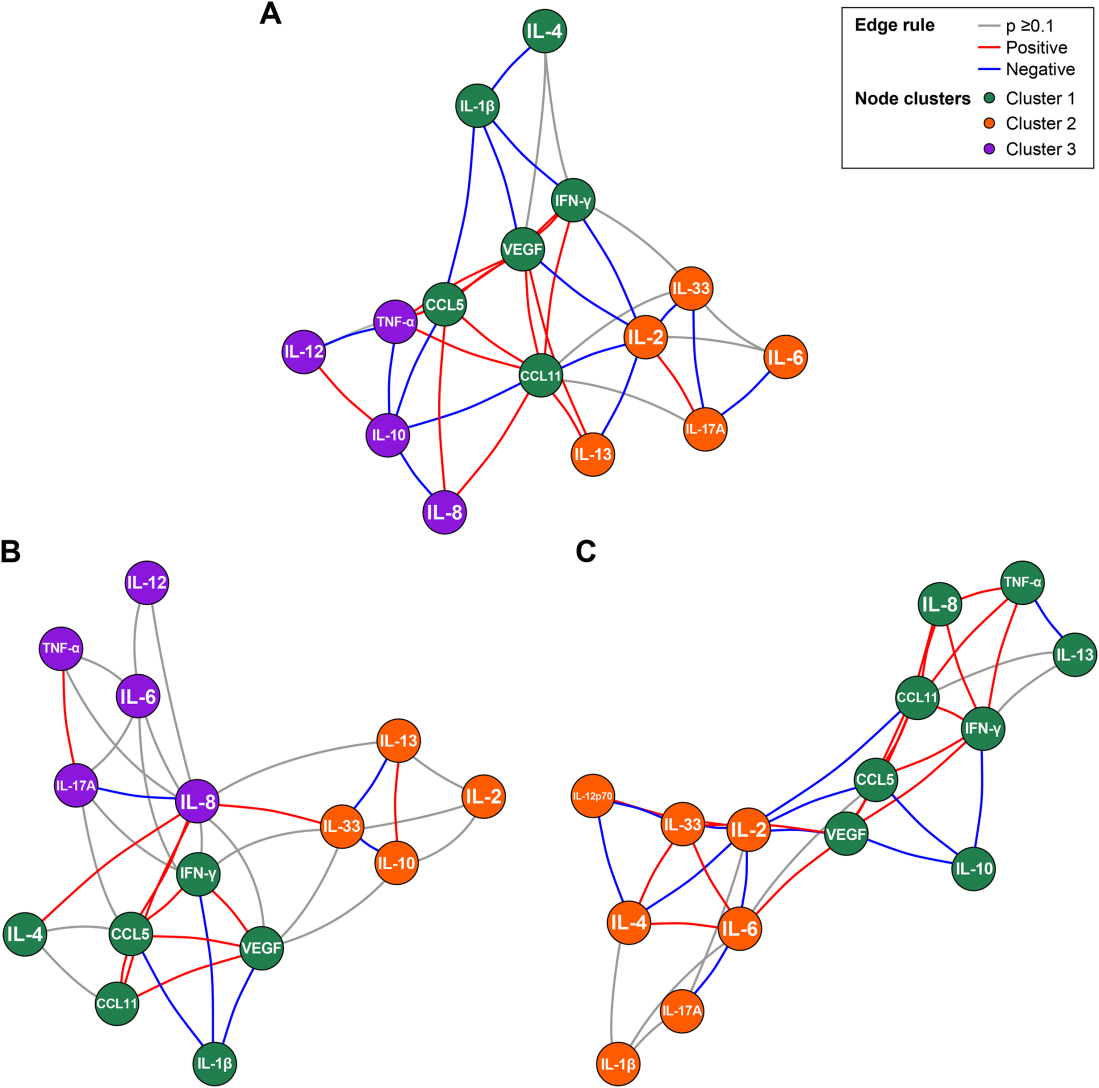
Exploratory graph analysis of baseline cytokine network structures in early and well-controlled (EW), late or partly controlled (LP), and nonresponder (NR) groups. Network structures were constructed using centered log-ratio (CLR)-transformed cytokine values. Partial correlations were estimated with graphical least absolute shrinkage and selection operator, and immune communities were identified using the walktrap algorithm. Nodes (green = cluster 1; orange = cluster 2; purple = cluster 3) represent cytokines, and edges represent partial correlations (red = positive; blue = negative). Edge thickness indicates the strength of association (*P* < 0.1). **(A–C)** correspond to EW, LP, and NR groups, respectively.

EGA revealed distinct baseline cytokine network architectures across the EW, LP, and NR groups (**Figure 2**). Although the overall set of cytokines included in each network was similar, the pattern and direction of inter-cytokine associations differed markedly according to treatment response, as reflected by distinct configurations of red (positive) and blue (negative) edges within the colored cytokine clusters (gree, orange, and purple nodes).

In the EW group (**Figure 2A**), cytokines formed a well-organized, compartmentalized network. Regulatory cytokines exhibited inverse relationships with effector and inflammatory mediators. IL-10 showed negative correlations with IL-8, TNF-α, and CCL5, while IL-2 demonstrated inverse associations with IL-33, IFN-γ, CCL11, and IL-13. In addition, IL-6 and IL-17A were negatively correlated. Together, these features reflected a network characterized by coordinated counter-regulatory interactions between regulatory and effector cytokines.

In the LP group (**Figure 2B**), these inverse associations were attenuated. Negative correlations involving IL-2 were largely absent, and IL-10 displayed limited connectivity, showing an inverse association primarily with IL-33. In contrast, positive correlations emerged among inflammatory mediators, including IL-8 with IL-33, IL-4, and CCL5, indicating partial reorganization of cytokine interactions.

In the NR group (**Figure 2C**), negative correlations between regulatory and effector cytokines were largely lost. Instead, positive associations predominated, particularly among IL-33, IL-4, and IL-6, suggesting a network structure dominated by effector and alarmin-related cytokines with minimal regulatory counterbalance.

Collectively, these findings demonstrate that baseline cytokine network topology differs substantially across omalizumab response groups, with progressively reduced regulatory-effector antagonism and increasing dominance of effector cytokine interactions from EW to NR patients.

### 3.4 Cytokine predictors distinguishing omalizumab response groups

**Figure 3:**
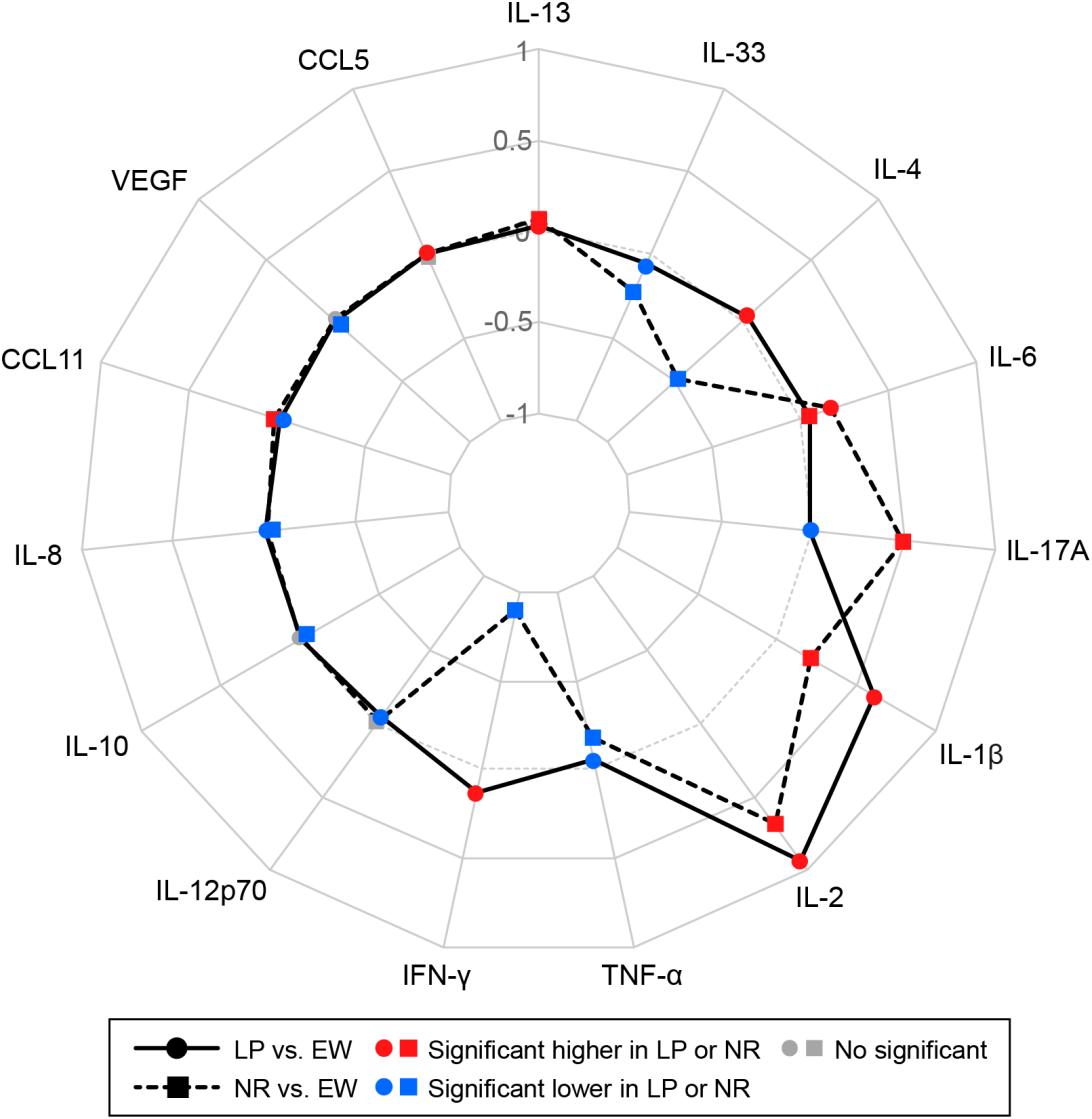
Radar plot comparing baseline cytokine differences among treatment response groups. Baseline cytokines were evaluated as predictors of treatment response using multinomial logistic regression, with early and well-controlled (EW) as the reference. The radar plot displays log_10_-transformed odds ratios for late or partly controlled (LP; solid line with • markers) and nonresponder (NR; dashed line with ◼ markers) groups. Values above or below zero indicate higher or lower odds compared to EW. Cytokines with significant differences (*P* < 0.05) are highlighted (red = higher odds; blue = lower odds).

Multinomial logistic regression identified several baseline cytokines that differentiated omalizumab response groups (**Figure 3**). A graded pattern was observed across EW to LP to NR, with IL-13, IL-6, IL-1β, and IL-2 demonstrating progressively higher odds ratios in poorer responders. Conversely, IL-33, IL-10, and TNF-α were associated with lower odds ratios in LP and NR compared to EW. Divergent patterns were noted for IL-4 and IFN-γ, which were elevated in LP but reduced in NR, whereas IL-17A levels were lower in LP but higher in NR, suggesting distinct cytokine configurations underlying partial versus complete non-response.

**Figure 4:**
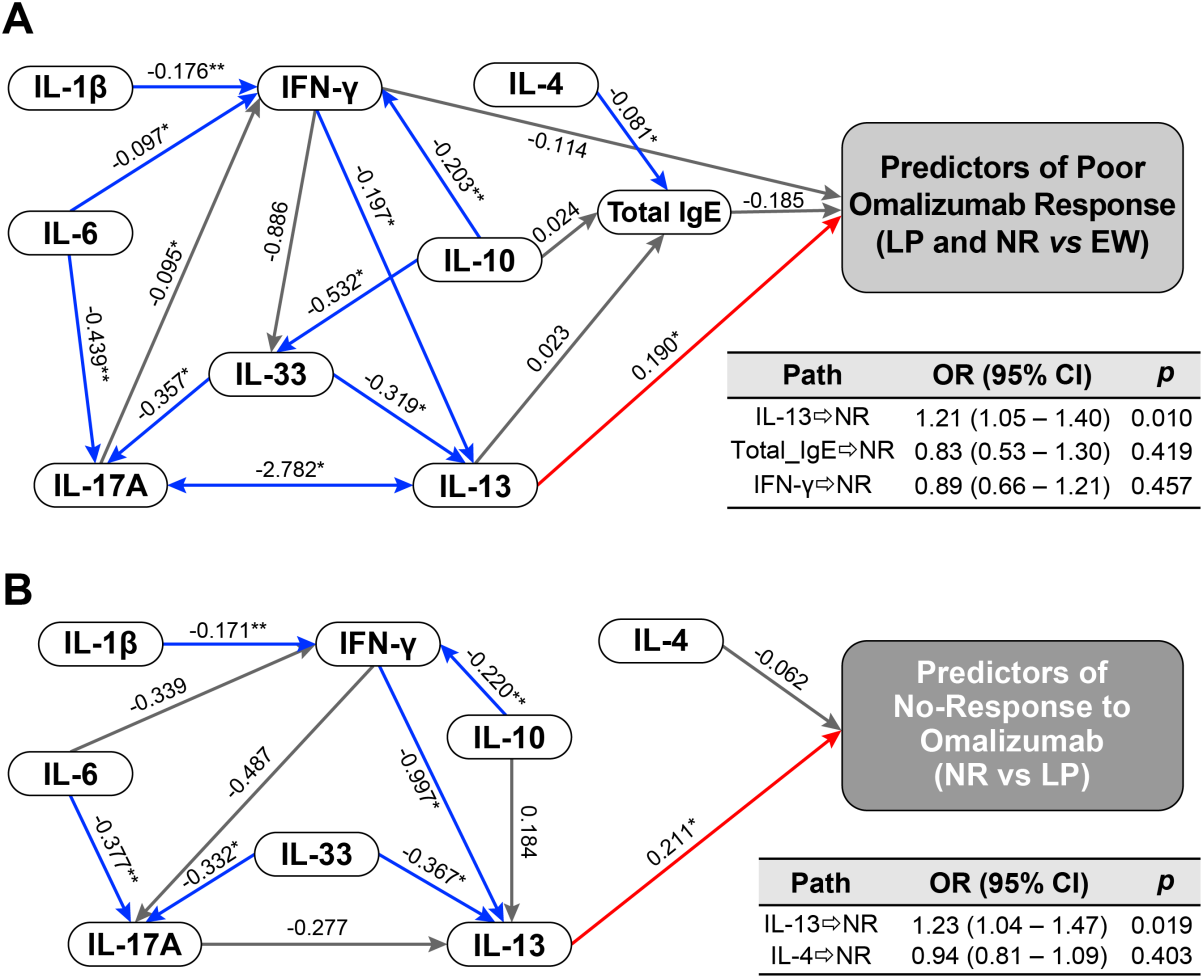
Structural equation modeling (SEM) of baseline cytokine network predictors of omalizumab treatment response. SEM incorporated centered log-ratio (CLR)-transformed baseline cytokines and total immunoglobulin E (IgE) levels. Model **(A)** identifies cytokine pathways associated with poor response (late or partly controlled [LP] + non-responder [NR]), whereas Model **(B)** identifies pathways distinguishing NR from LP. Fit indices: chi-square/degrees of freedom (χ²/df) = 1.024, *P* = 0.428; comparative fit index (CFI) = 0.959; root mean square error of approximation (RMSEA) = 0.019; standardized root mean square residual (SRMR) = 0.053 (Model A). Model B: χ²/df = 1.100, P = 0.352; CFI = 0.897; RMSEA = 0.039; SRMR displayed. Significance levels: *P < 0.05, **P < 0.01.

Structural equation modeling demonstrated generally acceptable model fit (χ²/df ≈ 1.0–1.1, CFI ≥ 0.90, RMSEA ≤ 0.04, SRMR ≈ 0.05; **Figure 4**). Across models, IL-13 consistently emerged as the primary cytokine directly associated with omalizumab response categories. Several additional cytokines, including IL-6, IL-1β, IL-33, IL-10, and IFN-γ, suggesting that multiple inflammatory signals shape response patterns through an IL-13-centered network. In the NR-specific model, IFN-γ and IL-33 showed negative paths toward IL-13-related pathways, suggesting attenuation of counter-regulatory or modulatory signals. IL-4 also exhibited an independent negative association with the NR group, implying that residual Th2 signaling may be linked to preserved responsiveness to omalizumab, although this effect did not reach statistical significance (**Figure 4B**).

Together, multinomial regression and SEM analyses identified IL-13 as a central cytokine underlying heterogeneity in omalizumab response, with distinct cytokine interaction patterns characterizing the LP and NR endotypes.

To further explore the relationship between cytokine profiles and conventional biomarkers, we assessed associations between IL-13, IL-17A, total IgE, and anti-TPO IgG. No significant correlations were observed between IL-13 or IL-17A and total IgE levels in the overall cohort. However, subgroup analyses suggested heterogeneous patterns, with a trend toward a negative correlation between baseline IL-17A and total IgE in LP patients (Spearman’s ρ = -0.462, *P* = 0.071), and a significant positive correlation between post-treatment IL-13 and total IgE in NR patients (ρ = 0.559, *P* = 0.025). In addition, longitudinal analysis indicated that IL-17A compositional changes over time differed according to anti-TPO IgG status (interaction β = -2.75, *P* = 0.013).

### 3.5 Changes in cytokines after 6 months of omalizumab treatment

**Figure 5:**
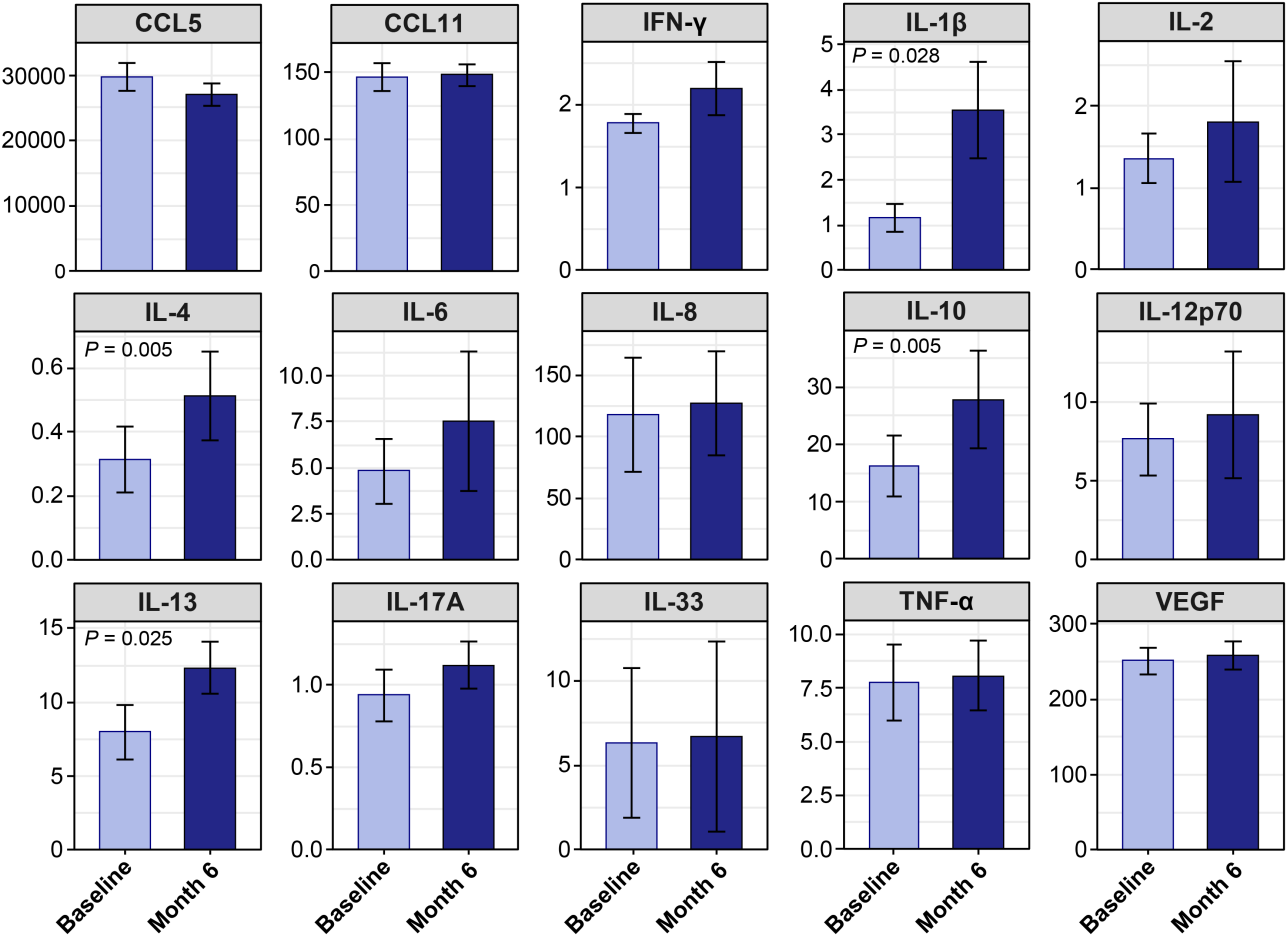
Cytokine levels at baseline and 6 months after omalizumab treatment. Mean cytokine concentrations (pg/mL, ± standard error of the mean) at baseline and at month 6 are shown for the total cohort. Within-patient comparisons were performed using the Wilcoxon signed-rank test. Significant differences (*P* < 0.05) are indicated above the corresponding bars.

When all patients were analyzed together, several cytokines showed significant changes after 6 months of omalizumab treatment (**Figure 5**). Serum levels of IL-1β, IL-4, IL-10, and IL-13 were significantly increased compared with baseline.

**Figure 6:**
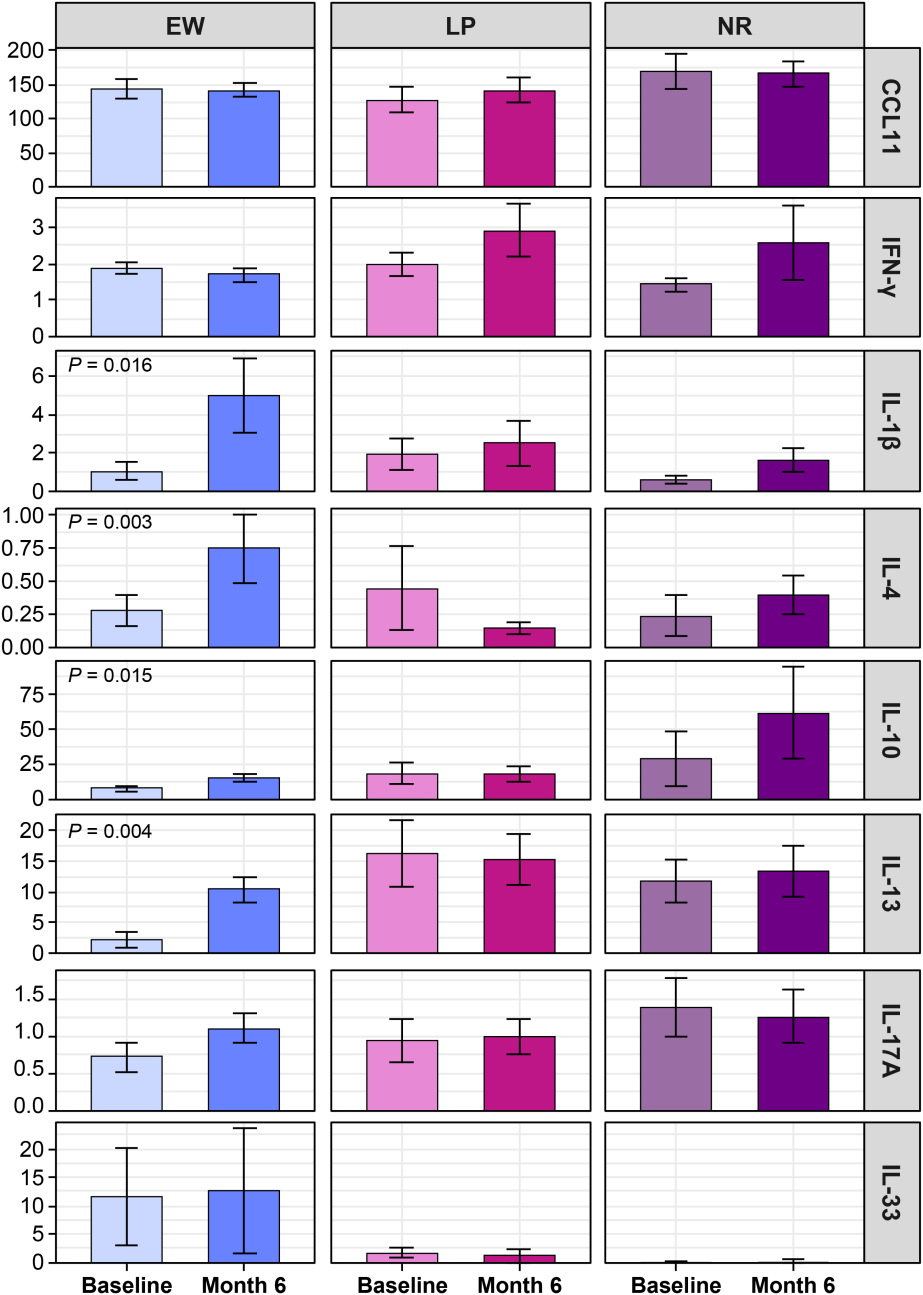
Within-patient cytokine changes from baseline to 6 months by omalizumab response subgroups. Cytokine concentrations (pg/mL, ± standard error of the mean) at baseline and at month 6 are shown separately for early and well-controlled (EW), late or partly controlled (LP), and nonresponder (NR) groups. Within-group comparisons were performed using the Wilcoxon signed-rank test. Significant changes (*P* < 0.05) are indicated within each panel.

When stratified by treament response, within-patient cytokine changes differed across groups (**Figure 6**, **Supplementary Table 1**). The significant increases observed at the cohort level, namely IL-1β, IL-4, IL-10, and IL-13, were detected only in the EW group. In contrast, the LP and NR groups did not exhibit significant longitudinal cytokine changes over 6 months.

Overall, measurable cytokine remodeling during omalizumab treatment was confined to EW patients, whereas LP and NR groups exhibited minimal or no longitudinal cytokine changes.

## 4 Discusion

In this study, we demonstrate that heterogeneity in omalizumab response among patients with H1AH-refractory CSU is closely linked to baseline cytokine composition and network organization rather than to absolute cytokine concentrations alone. By integrating compositional cytokine analysis, network-based interaction profiling, multivariate modeling, and longitudinal assessment, we show that an IL-13-dominant cytokine configuration with limited regulatory flexibility characterizes patients with delayed, partial or absent responses to omalizumab. In contrast, EW responders exhibited a more proportionate and flexible cytokine architecture at baseline and demonstrated measurable cytokine modulation over 6 months of treatment, whereas LP and NR patients maintained largely unchanged inflammatory profiles. These findings suggest that baseline inflammatory architecture may critically influence the capacity of anti-IgE therapy to modulate downstream immune pathways in CSU.

Previous studies have reported heterogeneous cytokine patterns in CSU, involving Th2, Th1, Th17, and innate pathways (13–15, 18). However, most studies have focused on individual cytokines or absolute concentrations, resulting in inconsistent findings and limited mechanistic insight (15, 19–22). A longitudinal study reported modest changes in IL-4 and IL-5 levels during omalizumab therapy but did not stratify patients by clinical response (20). Together, these observations indicate the limitations of single-cytokine approaches in explaining treatment-response heterogeneity. Our findings extend this literature by demonstrating that a compositional and network-based framework more clearly distinguishes clinical response trajectories.

In the present study, IL-13 was the only cytokine differing significantly among response groups when assessed as a raw concentration. However, compositional analysis revealed an IL-13-dominant imbalance as a defining feature of poor responders. This emphasizes the importance of relative cytokine balance over isolated cytokine measurements and suggests that IL-13 predominance reflects a broader immunologic state rather than a simple quantitative excess. Notably, while LP and NR patients shared an IL-13-dominant cytokine background with attenuation of modulatory and counter-regulatory cytokines, their secondary cytokine configurations diverged. LP patients retained partial Th2 and Th1 features, whereas NR patients exhibited further deviation characterized by lower baseline IL-4 and IFN-γ levels, indicating biologically distinct endotypes underlying partial versus complete nonresponse.

These findings further suggest that cytokine-network dynamics may not be fully captured by conventional biomarkers such as total IgE or anti-TPO autoantibodies. The lack of consistent correlations in the overall cohort, together with subgroup-specific associations, highlights the heterogeneity of underlying inflammatory mechanisms and supports the need for complementary endotype stratification approaches beyond classical biomarkers.

Network-based analysis further refined this interpretation. In EW patients, regulatory cytokines such as IL-10 and IL-2 were integrated with Th2, Th1, and innate effector pathways, consistent with a dynamically regulated immune network. In contrast, LP and NR patients showed progressive disruption of these regulatory-effector interactions, with reinforcement of innate and chemokine-associated modules. Such network rigidity likely constrains the downstream immunologic impact of IgE neutralization, providing a mechanistic explanation for persistent disease activity despite adequate pharmacologic exposure.

Reduced IL-33 relative to IL-13 in LP and NR patients may reflect a later, post-alarmin phase of CSU inflammation. IL-33 is an epithelial alarmin that initiates type 2 immune responses and primes type 2 innate lymphoid cells (ILC2) and mast cells (21–24). Once established, however, ILC2-driven IL-13 production can persist independently of ongoing epithelial IL-33 release (23, 24). Consistent with this model, our findings suggest that LP and NR patients may represent a chronically primed type 2 endotype characterized by persistent IL-13 activity despite attenuated upstream alarmin signals. These interpretations require validation in future studies incorporating receptor-level and tissue-based analyses. Importantly, IL-13 predominance in this context may reflect a partially IgE-independent type 2 circuit that limits responsiveness to anti-IgE therapy.

Multivariate pathway modeling consistently positioned IL-13 as a central integrator of treatment-response heterogeneity. While IL-13 showed the strongest direct association with response categories, multiple cytokines, including IL-6, IL-1β, IL-33, IL-10, IFN-γ, and IL-17A, contributed indirectly by shaping IL-13-related pathwaysThese findings imply that residual Th2 signaling may be linked to preserved responsiveness even in late responders. Although these models cannot establish causality, they support the concept that diverse inflammatory inputs converge toward an IL-13-skewed, biologically inflexible configuration that limits the effectiveness of IgE-targeted therapy.

Longitudinal Findings further support reduced immune plasticity in LP and NR patients. Although not all differences reached statistical significance, relatively sustained levels of IL-17A and CCL11 in NR patients after treatment are in line with prior studies implicating innate-Th17-skewed inflammation in chronic, severe, and H1AH-refractory CSU (13, 18, 25). IL-17A can activate keratinocytes, endothelial cells, and innate immune cells independently of IgE, providing plausible mechanisms for persistent symptoms despite anti-IgE therapy (26, 27). The disease is increasingly recognized as involving coordinated interactions among mast cells, basophils, eosinophils, T cells, ILC2, and natural killer cells through cytokines, chemokines, lipid mediators, and direct cell-cell communication (15, 28–30).

Within this framework, CCL11, a key chemokine for eosinophil trafficking, may function as a downstream effector of type 2 inflammation rather than a primary driver. In our cohort, CCL11 levels remained relatively elevated after 6 months of omalizumab treatment in LP and NR patients, paralleling the lack of cytokine remodeling. This pattern is compatible with a biologically rigid type 2 effector milieu characterized by IL-13 predominance and limited longitudinal modulation, which may be insufficiently controlled by IgE blockade alone.

These findings have potential translational implications. Patients with IL-13-dominant endotypes may benefit from therapies targeting IL-4Rα or IL-13 blockade, whereas those with features of innate or Th17-skewed profiles may require agents targeting IL-17, IL-1β, IL-23, or Bruton’s tyrosine kinase (25, 31–33). These hypotheses require validation in prospective studies.

This study has limitations. First, it was conducted at a single center with a modest sample size. Second, the cytokine panel included only 15 analytes and did not capture other relevant mediators, including alarmins, complement components, proteases, or neuroimmune factors. Third, functional assays for type I or type IIb autoimmune endotypes were not comprehensively evaluated. Fourth, although SEM provides insight into pathway-level associations, it cannot establish causal relationships. Accordingly, larger, multicenter studies incorporating expanded immunologic profiling and functional assays will be necessary to validate these observations.

In conclusion, this study demonstrates that distinct cytokine composition patterns and immune-network features, most notably IL-13 predominance within an attenuated modulatory context, are associated with incomplete or absent responses to omalizumab in H1AH-refractory CSU. Despite 6 months of treatment, LP and NR patients showed minimal cytokine remodeling, reflecting a biologically rigid inflammatory state. Notably, conventional biomarkers, including total IgE and anti-TPO IgG, did not clearly distinguish response groups. Compared with EW responders, NR patients were further characterized by lower baseline IL-4 and IFN-γ levels, together with relatively higher IL-17A levels, delineating a distinct inflammatory configuration that was not modified by omalizumab therapy. Collectively, these findings provide a conceptual framework for endotype-guided treatment strategies in CSU and support the integration of cytokine profiling with established clinical and functional biomarkers to optimize biologic selection and long-term disease control.

## Data Availability

The datasets presented in this article are not readily available because As the data were not collected with informed consent specifically permitting secondary sharing, it would be difficult in principle to anonymize and share the data. Requests to access the datasets should be directed to ye9007@ajou.ac.kr.
