# Supplementary material for: Cytokine imbalance and immune network endotypes underlying heterogeneous response to omalizumab in chronic spontaneous urticaria
Source: Front Immunol. 2026 Apr 20;17:1783483. doi: 10.3389/fimmu.2026.1783483 (PMC13135958; doi:10.3389/fimmu.2026.1783483)
Supplement: Supplementary file 1 [file Table1.docx]

Supplementary Table S1. Baseline levels and change-from-baseline values of 15 serum cytokines at month 6 of omalizumab treatment

| Cytokine levels (pg/mL) | | EW group  (n=33) | LP group  (n=16) | NR group  (n=16) | *P* value |
| --- | --- | --- | --- | --- | --- |
| Baseline | CCL11 | 144.49±76.79 | 127.33±75.44 | 169.24±100.30 | 0.476 |
|  | IFN-γ | 1.85±0.86 | 1.96±1.25 | 1.44±0.71 | 0.299 |
|  | IL-1β | 1.04±2.46 | 1.93±3.30 | 0.64±0.79 | 0.141 |
|  | IL-2 | 0.84±1.62 | 2.00±2.73 | 1.78±3.16 | 0.118 |
|  | IL-4 | 0.28±0.68 | 0.45±1.27 | 0.24±0.61 | 0.450 |
|  | IL-6 | 4.55±13.64 | 6.97±18.77 | 3.21±8.77 | 0.324 |
|  | IL-8 | 144.03±431.89 | 164.27±429.43 | 17.40±19.96 | 0.104 |
|  | IL-10 | 8.32±14.38 | 19.15±29.75 | 29.41±79.02 | 0.087 |
|  | IL-12 | 4.27±7.64 | 5.72±7.05 | 16.41±34.50 | 0.220 |
|  | IL-13 | 2.26±6.79 | 16.13±21.86 | 11.73±14.50 | 0.003 |
|  | IL-17A | 0.72±1.14 | 0.94±1.14 | 1.38±1.59 | 0.278 |
|  | IL-33 | 11.62±49.68 | 1.60±3.49 | 0.07±0.22 | 0.267 |
|  | CCL5 (*1000) | 24.81±11.13 | 40.44±21.39 | 29.01±17.85 | 0.051 |
|  | TNF-α | 9.20±19.31 | 6.15±7.38 | 6.30±3.82 | 0.595 |
|  | VEGF | 246.79±137.01 | 293.19±193.21 | 217.28±107.57 | 0.629 |
| Change from baselines | CCL11 | 142.40±57.06 | 142.26±70.55 | 165.57±72.73 | 0.619 |
|  | IFN-γ | 1.69±1.06 | 2.88±2.82 | 2.56±4.02 | 0.267 |
|  | IL-1β | 4.99±11.28 | 2.50±4.80 | 1.60±2.47 | 0.190 |
|  | IL-2 | 1.76±7.15 | 1.22±3.05 | 2.47±5.48 | 0.799 |
|  | IL-4 | 0.75±1.47 | 0.14±0.17 | 0.40±0.58 | 0.163 |
|  | IL-6 | 11.89±42.22 | 2.03±1.69 | 4.00±6.40 | 0.237 |
|  | IL-8 | 191.70±459.73 | 48.99±53.10 | 72.07±129.72 | 0.830 |
|  | IL-10 | 15.68±14.36 | 18.71±23.49 | 61.82±130.76 | 0.399 |
|  | IL-12 | 4.53±7.80 | 6.43±6.98 | 21.50±63.18 | 0.286 |
|  | IL-13 | 10.35±11.94 | 15.35±16.58 | 13.31±16.04 | 0.112 |
|  | IL-17A | 1.11±1.11 | 1.00±0.93 | 1.26±1.41 | 0.540 |
|  | IL-33 | 12.48±63.41 | 1.07±4.27 | 0.30±1.20 | 0.855 |
|  | CCL5 (*1000) | 25.08±12.62 | 32.32±18.61 | 25.64±10.12 | 0.073 |
|  | TNF-α | 9.95±15.43 | 3.39±3.97 | 8.79±13.08 | 0.413 |
|  | VEGF | 262.60±134.52 | 257.90±182.80 | 248.20±154.77 | 0.132 |

EW, early and well-controlled; LP, late or partly controlled; NR, non-responder

Values are mean ± standard deviation. *P* values refer to comparisons across EW, LP, and NR groups (Kruskal–Wallis test).
